# Photonic Generation of Arbitrary Microwave Waveforms with Anti-Dispersion Transmission Capability

**DOI:** 10.3390/mi15101214

**Published:** 2024-09-29

**Authors:** Xinyan Zhang, Kunpeng Zhai, Sha Zhu, Huashun Wen, Yu Liu, Ninghua Zhu

**Affiliations:** 1 Key Laboratory of Optoelectronic Materials and Devices, Institute of Semiconductors, Chinese Academy of Sciences, Beijing 100083, China; zhangxinyan@semi.ac.cn (X.Z.); yliu@semi.ac.cn (Y.L.); 2 University of Chinese Academy of Sciences, Beijing 100049, China; 3 Institute of Intelligent Photonics, Nankai University, Tianjin 300071, China; whs@semi.ac.cn (H.W.); nhzhu@semi.ac.cn (N.Z.); 4 College of Electronic Information and Optical Engineering, Nankai University, Tianjin 300071, China

**Keywords:** microwave photonics, waveform generation, anti-dispersion transmission

## Abstract

We propose and demonstrate a photonic-assisted approach for generating arbitrary microwave waveforms based on a dual-polarization dual-parallel Mach–Zehnder modulator, offering significant advantages in terms of tunability of repetition rates and anti-dispersion capability. In order to generate diverse microwave waveforms, two sinusoidal radio frequency signals with distinct frequency relationships are applied to the dual-polarization dual-parallel Mach–Zehnder modulator. By adjusting the power of the applied sinusoidal radio frequency signal, the power ratio between these orthogonal polarized optical sidebands can be changed, and thereby desired radio frequency waveforms can be obtained after photoelectric conversion. In our proof-of-concept experiment, we systematically varied the repetition rate of triangular, rectangular and sawtooth waveforms. Meanwhile, we calculated the Root Mean Square Error (RMSE) to assess the approximation error in each waveform. The RMSEs are 0.1089, 0.2182 and 0.1185 for the triangular, rectangular and sawtooth microwave waveforms with repetition rate of 8 GHz, respectively. Furthermore, after passing through 25 km single mode fiber, the optical power decreased by approximately 5.6 dB, which verifies the anti-dispersion transmission capability of our signal generator.

## 1. Introduction

There has been a recent surge in interest in the field of photonics for the generation of arbitrary microwave waveforms, showing its potential for various crucial applications in contemporary radar systems, wired and wireless communication networks, as well as microwave photonics systems [1,2,3,4,5]. In the domain of optical frequency conversion, pulse compression, and signal replication, triangular and rectangular waveforms are used extensively in comparison to alternative signal profiles [6,7,8]. However, the frequency and bandwidth of conventional electrical microwave waveform generators are limited by the electron bottleneck. Photonic techniques exhibit substantial promise, owing to their rich spectrum resources in the optical domain, high frequency, large bandwidth, ultra-low transmission loss, anti-electromagnetic interference and flexible tuning and reconfiguration, which is a promising candidate to overcome the electron bottleneck. Optoelectronic devices can further improve the system performance and reduce power consumption compared with electrical devices.

Several photonic methods have been reported for the generation of arbitrary microwave waveforms. A commonly employed approach involves integrating optical spectral shaping with frequency-to-time mapping [9,10]. An optical pulse generated through a mode locked laser (MLL) is tailored using an optical spectral shaper to obtain a desired microwave waveform by directing the tailored optical signal to a photodetector (PD) [9]. In [10], the optical spectrum is transformed into a triangular shape by cascading two sinusoidal filter modules. Periodic triangular waveforms with the same shape as the optical spectrum are generated through frequency-to-time conversion in a dispersive fiber. However, the radio frequency (RF) waveforms generated using optical spectra shaping are typically characterized by a small duty cycle and lack of flexibility.

Furthermore, microwave waveforms can also be generated using external modulation. The nonlinearity of the external modulator leads to the generation of optical sidebands. By manipulating the phases and amplitudes of these optical sidebands, the desired RF waveforms can be generated. For instance, the rectangular and triangular waveforms can be generated through the utilization of a modulator in conjunction with a waveshaper, which is used to obtain the desired sidebands [11]. However, the bandwidth of the system is limited. Dispersive fiber also can be used for suppressing undesired harmonics. However, the tunability of the repetition rate is poor due to the need for adjusting the length of dispersive fiber in accordance with changes in the frequency of the RF signal [12]. In [13], triangular or rectangular microwave signals with tunable duty cycle can be generated based on a single-drive modulator and a tunable optical delay line. A stimulated Brillouin scattering effect in the optical fiber also can be utilized to generate a full-duty-cycle triangular waveform, but the sensitivity of the SBS effect to environmental fluctuations may introduce instability into the system [14]. Moreover, previous works have demonstrated the use of two polarization-dependent MZMs [15] or a polarization modulator (PolM) in a Sagnac loop [16] to generate triangular and rectangular waveforms, but the structure is relatively complicated and costly. It is noted that previous works normally suffer from limitations such as small duty cycle, difficulties in tuning the repetition rate or a complicated system. Moreover, the utilization of a frequency multiplier or a 90-degree electrical hybrid coupler is not conducive to practical applications. However, none of the above works assesses and verifies the anti-dispersion capability of the signal, and so they are unable to achieve anti-dispersion transmission. Long-fiber transmitting is essential in applications such as pulse shaping, radar networks, optical communication, fiber sensing and signal processing. In multi-static radar networks, long-distance fiber transmission from the central office (CO) to base stations (BS) introduces frequency-dependent phase shifts. These shifts can result in significant periodic power fading at certain frequencies, potentially degrading system performance. Therefore, the further investigation into anti-dispersion transmission of microwave waveforms is necessary.

In this work, we proposed and experimentally demonstrated a photonically assisted method to generate arbitrary microwave waveforms with tunable repetition rates. Furthermore, the anti-dispersion transmission capability of the system is also comprehensively studied. The main structure is based on one dual-polarization dual-parallel Mach–Zehnder modulator (DP-DPMZM). The generation procedure can be regarded as the composition and beating of limited optical spectrum terms. It is worth noting that we can generate a wide variety of waveforms beyond the basic triangular, sawtooth, and rectangular waves, including ramp waves, sinusoidal combinations, modulated waves, beating signals, pulse waves, aperiodic waveforms. Moreover, each of these processes has applications in signal processing, communication, acoustics. It is known that the Fourier series expansion of the ideal triangular waveform exclusively consists of odd-order harmonics, which can be effectively implemented by employing sinusoidal signals to modulate a light wave at a DP-DPMZM. By properly setting the DC bias of sub-MZMs and manipulating the power of RF input signals, desired triangular, rectangular, and sawtooth waveforms can be obtained. The microwave waveforms can be well reconfigured by adjusting the RF frequency while maintaining a full-duty cycle. In our experimental setup, the repetition rate of triangular, rectangular, and sawtooth waveforms can be adjusted within the frequency range of 8 to 12 GHz. In addition, carrier-suppressed single-sideband (CS-SSB) modulation is the key to achieve long-fiber transmission. Anti-dispersion transmission capability is verified by inserting a 25 km SMF into the system link, and the waveform quality remains excellent.

## 2. Principle

**Figure 1:**
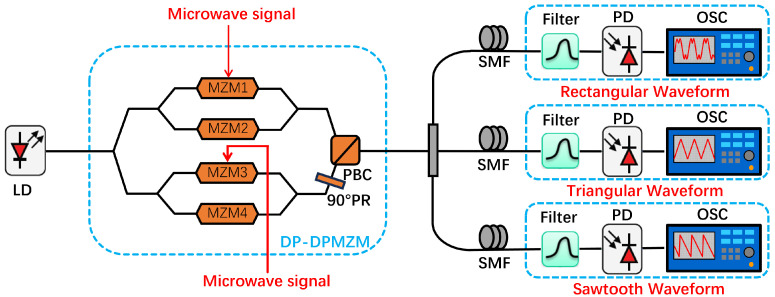
Schematic diagram of the proposed photonics generation of microwave waveforms. LD: laser diode, MZM: Mach–Zehnder modulator, PR: polarization rotator, PBC: polarization beam combiner, SMF: single-mode fiber, PD: photodetector, OSC: oscilloscope.

The schematic diagram of the proposed photonics generation of microwave waveforms is shown in Figure 1, which consists of a laser diode (LD), a DP-DPMZM, an optical filter and a photodetector (PD). The significant device is a DP-DPMZM, which is composed of an x-polarized dual-parallel MZM (*x*-DPMZM) and a y-polarized dual-parallel MZM (*y*-DPMZM). Specifically, the DP-DPMZM includes a polarization beam combiner (PBC), a 90° polarization rotator (PR) and four sub-MZMs, which are denoted as MZM1, MZM2, MZM3, and MZM4, respectively.

**Figure 2:**
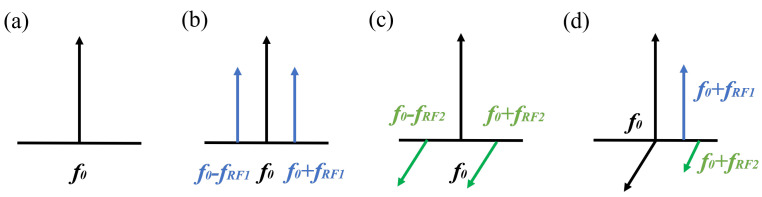
Schematic optical spectra of the proposed signal generator. (**a**) Optical carrier. (**b**) Optical carrier modulated by RF1 signal in x-polarization. (**c**) Optical carrier modulated by RF2 signal in y-polarization. (**d**) The optical spectrum at the output of tunable bandpass filter (TBPF).

The LD emits an optical carrier at a wavelength of 1550 nm, which is then injected into the DP-DPMZM. Two sinusoidal RF signals generated by two microwave signal generators (MGS) are applied to the DP-DPMZM. In detail, one is directed towards MZM1 while the other is directed towards MZM3. By appropriately setting MZM1 and MZM3 at minimum transmission point, two orthogonally polarized carrier-suppression double sideband (CS-DSB) modulated signals can be generated. Meanwhile, the DC bias-introduced phase shifts of MZM2 and MZM4 are set at 0. Considering the small signal modulation condition and employing Jacobi–Anger expansion, the optical field at the output of *x*-DPMZM and *y*-DPMZM can be mathematically expressed as

(1) $$\begin{array}{cc} E_{x-DPMZM}\left(t\right) & =E_{1}\left(t\right)+E_{2}\left(t\right)exp\left(j\theta_{x}\right)\\ \; & =\frac{1}{4}E_{c}\left(t\right)\left\{exp\left(j\beta_{1}sin\omega_{1}t\right)+exp\left[j\beta_{1}sin\left(\omega_{1}t+\pi \right)\right]exp\left(j\pi \right)\right\}\\ & +\frac{1}{4}E_{c}\left(t\right)exp\left(j\theta_{x}\right)\\ \; & =\frac{1}{4}E_{c}exp\left[j\left(\omega_{c}+\omega_{1}\right)t\right]J_{1}\left(\beta_{1}\right)+\frac{1}{4}E_{c}exp\left[j\omega_{c}t+j\theta_{x}\right] \end{array}$$

(2) $$\begin{array}{cc} E_{y-DPMZM}\left(t\right) & =E_{3}\left(t\right)+E_{4}\left(t\right)exp\left(j\theta_{y}\right)\\ \; & =\frac{1}{4}E_{c}\left(t\right)\left\{exp\left(j\beta_{2}sin\omega_{2}t\right)+exp\left[j\beta_{2}sin\left(\omega_{2}t+\pi \right)\right]exp\left(j\pi \right)\right\}\\ & +\frac{1}{4}E_{c}\left(t\right)exp\left(j\theta_{y}\right)\\ \; & =\frac{1}{4}E_{c}exp\left[j\left(\omega_{c}+\omega_{2}\right)t\right]J_{1}\left(\beta_{2}\right)+\frac{1}{4}E_{c}exp\left[j\omega_{c}t+j\theta_{y}\right] \end{array}$$

where $\omega_{c}$ and $E_{c}$ are the angular frequency and amplitude of the optical carrier; $\omega_{1}$ and $\omega_{2}$ are the angular frequency of the $RF_{1}$ signal and $RF_{2}$ signal; $V_{\pi 1}$ and $V_{\pi 2}$ refer to the half-wave voltage of the MZM1 and MZM3; $V_{1}$ and $V_{2}$ correspond to the DC biases of MZM1 and MZM3; $\beta_{1}$ = $\pi V_{1}/V_{\pi 1}$ and $\beta_{2}$ = $\pi V_{2}/V_{\pi 2}$ are the modulation index of the MZM1 and MZM3, which are controlled by the DC biases, $V_{1}$ and $V_{2}$; and $\theta_{x}$ and $\theta_{y}$ are the DC-introduced phase shifts of *x*-DPMZM and *y*-DPMZM, which are determined by the DC bias voltage applied to the *x*-DPMZM and *y*-DPMZM, respectively; $J_{1}$ is the 1st-order Bessel function of the first kind. According to Equations (1) and (2), the output optical signal of DP-DPMZM consists of optical carriers and a series of sidebands, which can be a simplified schematic of the optical spectrum shown in Figure 2a–c. Two +1st-order optical sidebands with inherently orthogonal polarization are generated since the DP-DPMZM is equipped with a polarization beam combiner (PBC) at its termination to combine two orthogonal polarization optical signals. Next, a tunable bandpass filter (TBPF) is connected after the modulator to eliminate the negative sidebands or the positive sidebands, as shown in Figure 2d. After photoelectric conversion, the recovered electrical signal can be given by

(3) $$\begin{array}{cc} i(t) & =R\cdot [i_{x}\left(t\right)+i_{y}\left(t\right)]\\ \; & =R\cdot [E_{x-DPMZM}\left(t\right)\cdot E_{x-DPMZM}{\left(t\right)}^{*}+E_{y-DPMZM}\left(t\right)\cdot E_{y-DPMZM}{\left(t\right)}^{*}]\\ \; & =\frac{1}{16}R\cdot E_{c}^{2}\left[J_{1}^{2}\left(\beta_{1}\right)+J_{1}^{2}\left(\beta_{2}\right)+2\right]+\frac{1}{8}R\cdot E_{c}^{2}J_{1}\left(\beta_{1}\right)\cdot cos\left(\omega_{1}t-\theta_{x}\right)\\ \; & +\frac{1}{8}R\cdot E_{c}^{2}J_{1}\left(\beta_{2}\right)\cdot cos\left(\omega_{2}t-\theta_{y}\right) \end{array}$$

where *R* is the PD responsivity. According to Equation (3), the electrical signal consists of two different frequency components.

Fourier series expansion is commonly used to represent a periodic signal as a combination of sine and cosine functions. Common waveforms, such as triangular, rectangular waves, and sawtooth waves, can be expressed by Fourier series expansion. They are composed of fundamental waves of different frequencies respectively. By choosing different series, the original waveform can be gradually approximated. The Fourier series expansion of a typical triangular waveform, a rectangular waveform and a sawtooth waveform are written as

(4) $$\begin{array}{c} T_{tr}\propto A_{tr}+B_{tr}\sum_{k=1,3,5}^{\infty }\frac{1}{k^{2}}cos\left(k\Omega t\right)\propto C_{tr}+cos\left(\Omega t\right)+\frac{1}{9}cos\left(3\Omega t\right) \end{array}$$

(5) $$\begin{array}{c} T_{sq}\propto A_{sq}+B_{sq}\sum_{k=1,3,5}^{\infty }\frac{1}{{\left(-1\right)}^{\frac{k-1}{2}}k}cos\left(k\Omega t\right)\propto C_{sq}+cos\left(\Omega t\right)-\frac{1}{3}cos\left(3\Omega t\right) \end{array}$$

(6) $$\begin{array}{c} T_{sa}\propto A_{sa}+B_{sa}\sum_{k=1}^{\infty }\frac{1}{k^{2}}sin\left(k\Omega t\right)\propto C_{sa}+sin\left(\Omega t\right)+\frac{1}{2}sin\left(2\Omega t\right) \end{array}$$

where $A_{tr}$, $B_{tr}$, $C_{tr}$, $A_{sq}$, $B_{sq}$, $C_{sq}$, $A_{sa}$, $B_{sa}$, $C_{sa}$ are constants; *k* is the order of the Bessel function; Ω is the angular frequency of the fundamental frequency. According to Equations (3) and (4), it is evident that the triangular waveform is only composed of odd-order harmonics. The even-order harmonics must be suppressed by biasing the MZM1 and MZM3 at the minimum transmission point. To the obtain triangular waveform, we manipulate the DC phase shifts of *x*-DPMZM $\theta_{x}$ and *y*-DPMZM $\theta_{y}$ to 2π. This ensures that the optical signal at the output of the TBPF includes an optical carrier and odd-order sidebands originating from MZM1 and MZM3. Additionally, the triangular waveforms can be well approximated by two Fourier components since the power of higher-order harmonics is insufficient. Therefore, it is essential to ensure that the value of the modulation index and the angular frequency of the RF signals should satisfy the following relationship:

(7) $$\begin{array}{c} \frac{J_{1}\left(\beta_{1}\right)}{J_{1}\left(\beta_{2}\right)}=\frac{1}{9};\frac{\omega_{1}}{\omega_{2}}=\frac{1}{3} \end{array}$$

Similarly, the rectangular waveform also exhibits only odd-order harmonics. In comparison to the generation of a triangular waveform, generating a rectangular waveform necessitates a greater number of Fourier series components to maintain an equivalent level of root mean square error (RMSE). As can be seen from Equation (6), a sawtooth waveform consists of both odd-order and even-order harmonics. The DC phase shifts of *x*-DPMZM $\theta_{x}$ and *y*-DPMZM $\theta_{y}$ are set at π/2. In our experimental demonstration, the input RF power is limited by the modulator, which leads us consider two Fourier series components. To generate ideal rectangular waveforms and sawtooth waveforms, Equations (8) and (9) should be satisfied, respectively.

(8) $$\begin{array}{c} \frac{J_{1}\left(\beta_{1}\right)}{J_{1}\left(\beta_{2}\right)}=-\frac{1}{3};\frac{\omega_{1}}{\omega_{2}}=\frac{1}{3} \end{array}$$

(9) $$\begin{array}{c} \frac{J_{1}\left(\beta_{1}\right)}{J_{1}\left(\beta_{2}\right)}=\frac{1}{2};\frac{\omega_{1}}{\omega_{2}}=\frac{1}{2} \end{array}$$

## 3. Experiment and Results

### 3.1. Experimental Setup

A proof-of-concept experiment was carried out to validate the proposed scheme. An optical carrier at 1550 nm, emitted from an LD, was injected into a commercial DP-DPMZM. Two microwave sources were used for modulation in the experiment to ensure the generation of different waveforms. A sinusoidal $RF_{1}$ signal generated by a microwave signal generator was applied to the MZM1, and a sinusoidal $RF_{2}$ signal was applied to the MZM3. The phase difference between $RF_{1}$ and $RF_{2}$ is zero. After passing through an optical bandpass filter, we acquired two distinct and orthogonal +1st-order sidebands, along with the optical carrier. By applying the optical signal to an optical spectrum analyzer with a resolution of 0.01 nm, the optical spectrum of the modulated optical signal was obtained. The output optical signal was detected utilizing a high-speed photodetector with 3 dB bandwidth of 70 GHz. Subsequently, the electrical signals produced by the photodetector were fed into both an electrical spectrum analyzer and an oscilloscope. The 40 GHz electrical spectrum analyzer was employed to measure the electrical spectra, while the oscilloscope showed the desired microwave waveforms, including triangular, rectangular, and sawtooth waveforms.

### 3.2. Waveform Generation

**Figure 3:**
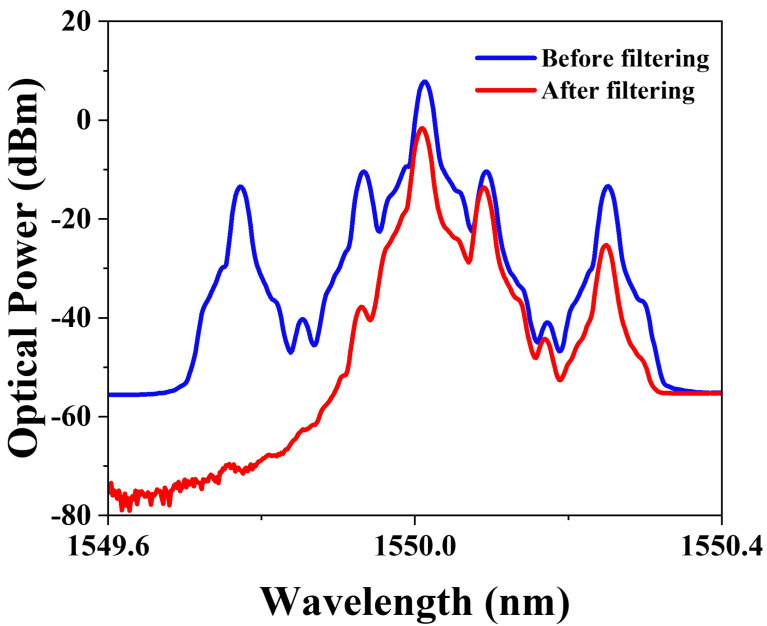
The measured optical spectra before and after the TBPF when generating a triangular waveform with a repetition of 10 GHz.

**Figure 4:**
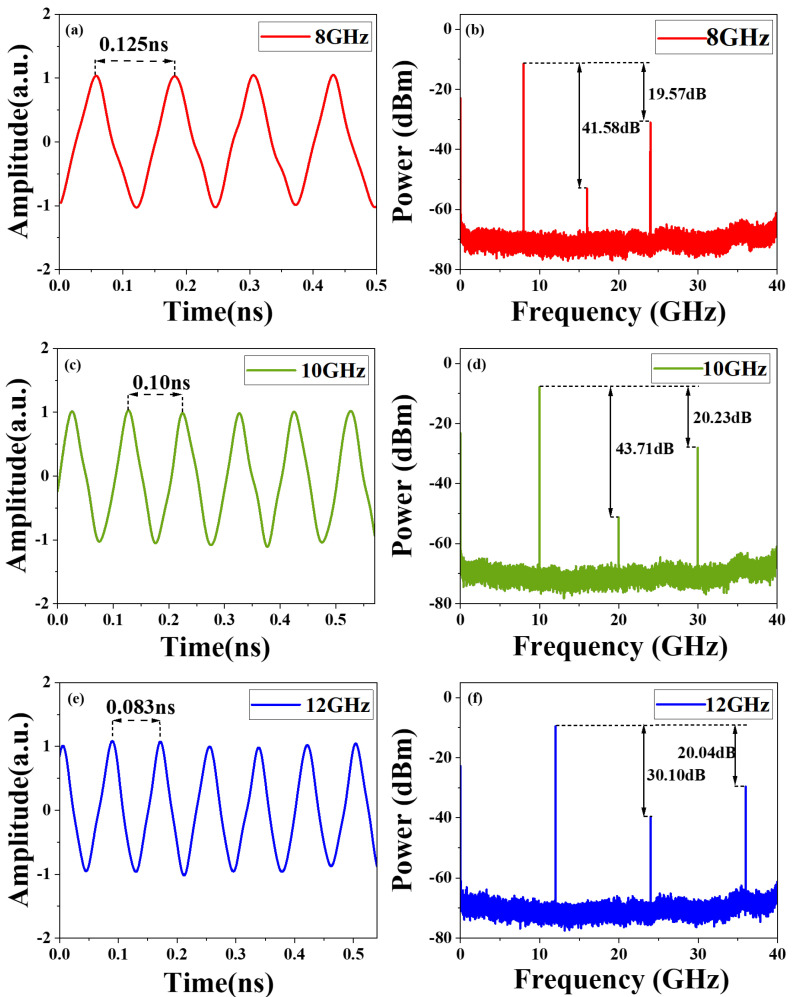
Experiment results. (**a**) Generated triangular waveform with a repetition rate of 8 GHz, and (**b**) its spectrum. (**c**) Generated triangular waveform with a repetition rate of 10 GHz, and (**d**) its spectrum. (**e**) Generated triangular waveform with a repetition rate of 12 GHz, and (**f**) its spectrum.

Firstly, an $RF_{1}$ microwave signal at 8 GHz was sent to MZM1, while a 24 GHz $RF_{2}$ microwave signal was applied to MZM3. MZM1 and MZM3 were all in a carrier-suppressed state by biasing them at minimum transmission points. The powers of RF signals were carefully controlled according to the Principle section. Figure 3 illustrates the measured optical spectrum at the output of the DP-DPMZM and optical bandpass filter. The corresponding electrical spectrum is depicted in Figure 4, where the most prominent features are the +1st-order harmonics at 8 GHz and 24 GHz. Since we do not use any electrical amplifiers or optical amplifiers, the final output electrical signal power is not high. In practical applications, power compensation can be used to achieve larger signal output. The power of the 24 GHz harmonic is 19.57 dB lower than that of the 8 GHz harmonic, closely aligning with the theoretical value of 19.08 dB. The 2nd-order harmonic at 16 GHz is effectively suppressed, exhibiting a difference of 41.48 dB compared to the first-order harmonic at 8 GHz. As shown in the waveform of Figure 4a, a triangular waveform with a repetition frequency of 8 GHz is successfully generated. To assess the approximation error in the waveform, we calculated the RMSE between the measured waveform and the ideal waveform. The RMSE of 0.1089 is close to the theoretical RMSE of 0.0482 calculated from the simulated waveform, and the discrepancy is primarily caused by the external environment and bias voltage instability, among other factors.

Our scheme has the potential for significant tunability and anti-dispersion ability. To further illustrate the tunability of the proposed waveform generation system, $RF_{1}$ microwave signals with center frequencies of 10 GHz and 12 GHz were loaded to *x*-DPMZM, respectively. The corresponding frequencies of the $RF_{2}$ microwave signals were set at 30 GHz and 36 GHz, respectively, satisfying the required frequency relationship for generating triangular waveforms. The measured electrical spectra and temporal waveforms are shown in Figure 4c–f. The power of the 30 GHz and 36 GHz harmonics are reduced by 20.23 dB and 20.04 dB, respectively, when compared to the 10 GHz and 12 GHz harmonics. Besides, the RMSE values between the measured and simulated rectangular waveforms are found to be 0.0827 and 0.1384, which fit well with the theoretical values 0.0484 and 0.0484, respectively.

**Figure 5:**
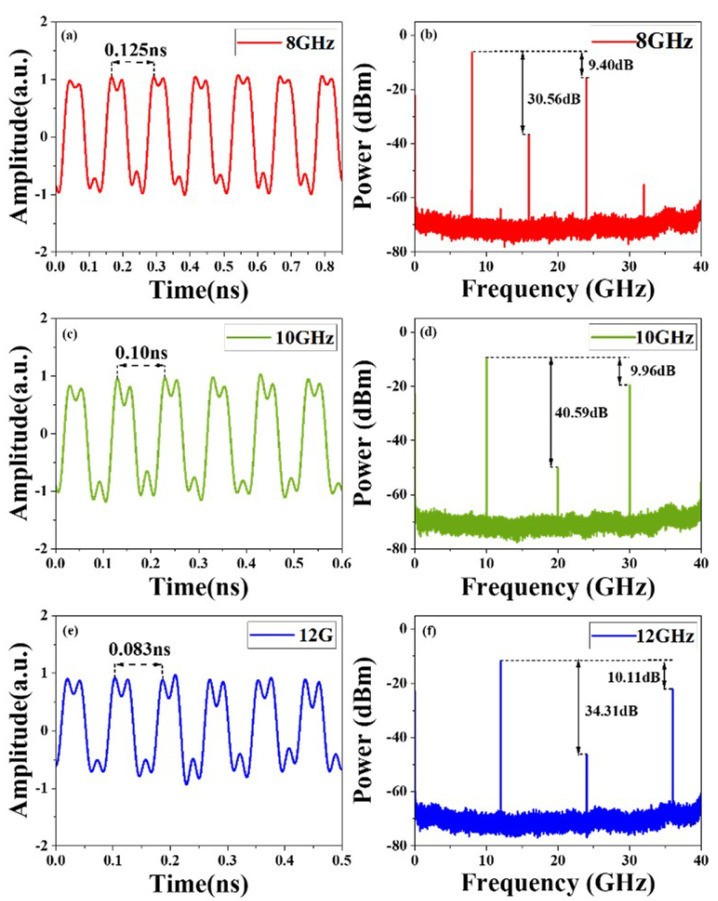
Experiment results. (**a**) Generated rectangular waveform with a repetition rate of 8 GHz, and (**b**) its spectrum. (**c**) Generated rectangular waveform with a repetition rate of 10 GHz, and (**d**) its spectrum. (**e**) Generated rectangular waveform with a repetition rate of 12 GHz, and (**f**) its spectrum.

As for the generation of rectangular waveforms, we successfully generated rectangular waveforms with tunable repetition rates. Again, the MZM1 was driven by 8, 10 and 12 GHz $RF_{1}$ microwave signals, while MZM3 was modulated by 24, 30 and 36 GHz $RF_{2}$ microwave signals. By biasing MZM1 and MZM3 at the minimum transmission point, the even-order sidebands of x-polarization and y-polarization were effectively suppressed. After filtering to remove undesired sidebands at lower wavelengths and photoelectric conversion, rectangular waveforms with repetition rates identical to the frequency of $RF_{1}$ microwave signals are generated, as shown in Figure 5a,c,e. When the $RF_{1}$ microwave signal was tuned at 8 GHz, it can be seen in Figure 5b that the power of the desired harmonic at 24 GHz is 9.40 dB lower than that of the 8 GHz harmonic, which is close to the theoretical one of 9.54 dB, and the undesired second-order harmonic at 16 GHz is 30.56 dB lower than first-order harmonic at 8 GHz. The RMSEs of rectangular waveforms were also calculated. For the rectangular waveforms at 8 GHz, 10 GHz, and 12 GHz, the RMSEs between measured waveforms with ideal waveforms were 0.2182, 0.2272 and 0.2507, which were close to the theoretical RMSEs of 0.2020, 0.2019, and 0.2017, calculated between ideal waveforms and simulated waveforms, respectively. To further improve the quality of the rectangular waveform, we can precisely adjust the phase of each harmonic component or utilize a higher sampling rate to capture more high-frequency information.

Subsequently, we demonstrated that the proposed waveform generator is capable of generating other waveforms, such as sawtooth waveforms. By applying an $RF_{1}$ microwave signal ranging from 8 GHz to 12 GHz to MZM1 and applying an $RF_{2}$ microwave signal with corresponding frequencies ranging from 16 GHz to 24 GHz to MZM3, the sidebands were precisely controlled to ensure that the frequency relationship complies with Equation (9). The power ratio between $RF_{1}$ and $RF_{2}$ microwave signals was carefully adjusted to satisfy Equation (9). Both MZM1 and MZM3 were biased at minimum transmission points while ensuring that the DC-introduced phase shifts of *x*-DPMZM and *y*-DPMZM, $\theta_{x}$ and $\theta_{y}$ were set at π/2. Thus, an optical signal with two orthogonal polarized sidebands and optical carrier was obtained.

**Figure 6:**
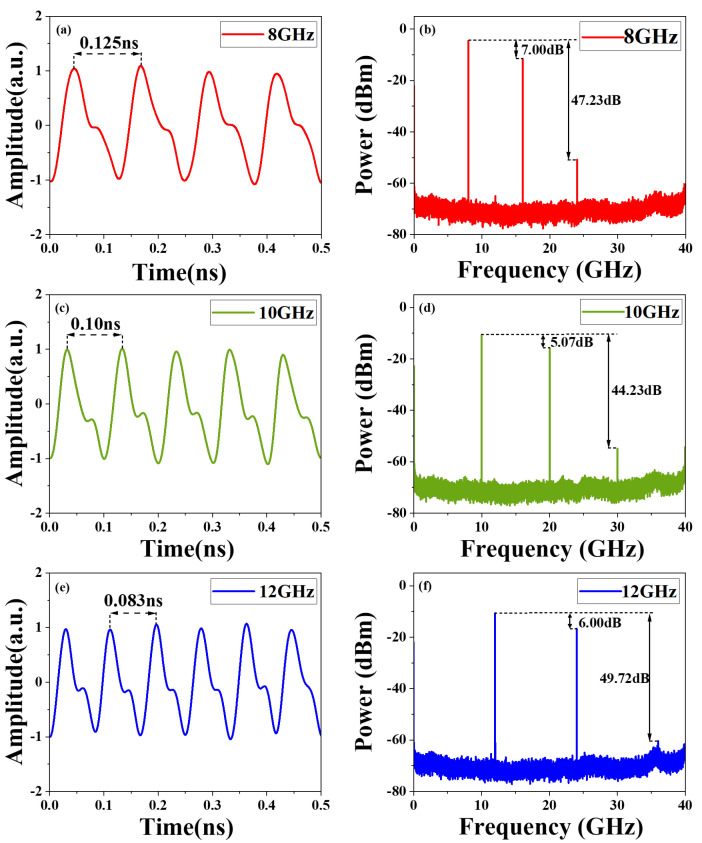
Experiment results. (**a**) Generated sawtooth waveform with a repetition rate of 8 GHz, and (**b**) its spectrum. (**c**) Generated sawtooth waveform with a repetition rate of 10 GHz, and (**d**) its spectrum. (**e**) Generated sawtooth waveform with a repetition rate of 12 GHz, and (**f**) its spectrum.

In the experiment, sawtooth waveforms with repetition rates identical to the frequency of $RF_{1}$ microwave signals were generated, as shown in Figure 6a,c,e. The corresponding electrical spectrums of the sawtooth waveforms are shown in Figure 6b,d,f. For the sawtooth waveform at 8 GHz, the electrical signals primarily consist of the fundamental tone at 8 GHz and the harmonic at 16 GHz, whose amplitude is 7 dB higher than the fundamental frequency. The contribution from the harmonic at 24 GHz can be neglected, as it is attenuated by 47.23 dB, compared to the fundamental frequency. The RMSEs are 0.1185, 0.1576 and 0.1323 for the sawtooth waveforms with 8, 10 and 12 GHz repetition rates, respectively.

### 3.3. Anti-Dispersion Transmission

**Figure 7:**
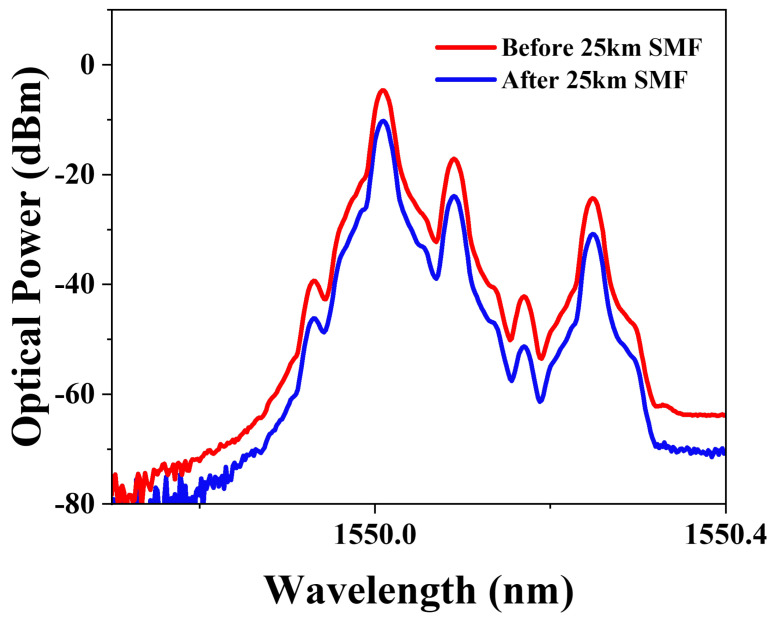
The measured optical spectra before and after 25 km SMF when generating a triangular waveform with the repetition of 10 GHz.

We also verified the anti-dispersion transmission ability of the system. In practical microwave photonic radar applications, different waveforms correspond to different applications. Rectangular signals are commonly used in time-division multiplexing (TDM) systems and digital modulation. Triangular signals are often employed in coherent detection, optical frequency scanning and modulator linearization. And sawtooth signals are widely used in applications such as frequency sweeping, as well as in certain fiber-optic sensing scenarios. This system is suitable for a variety of microwave photonic radar processing systems. In previous work, most photonic generation of microwave waveforms relied on double-sideband modulation, which suffers from power fading during long transmission. The method we proposed is based on carrier-suppressed single-sideband (CS-SSB) modulation. To demonstrate the anti-dispersion transmission and integrity of the waveform after passing the system through 25 km of optical fiber with 5.5 dB loss, we measured the optical spectrum before and after transmission through 25 km single-mode fiber (SMF), as displayed in Figure 7. It can be seen that there is a power decline when the link includes the 25 km SMF. The optical power attenuated by approximately 5.6 dB.

**Figure 8:**
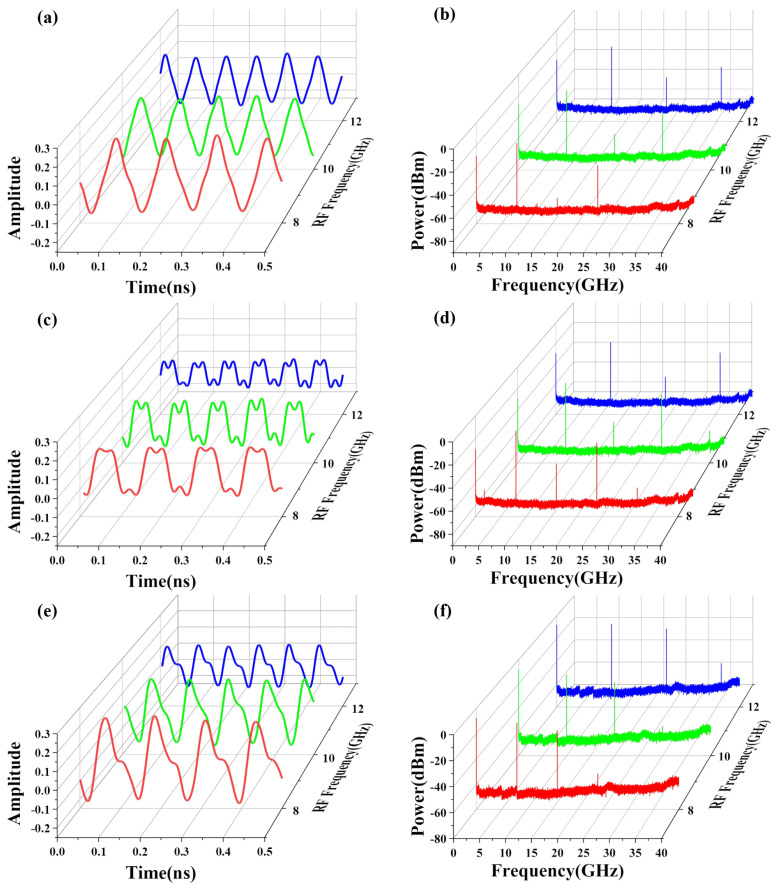
Experiment results. (**a**) Generated rectangular waveforms with repetition rate of 8, 10 and 12 GHz after 25 km SMF, and (**b**) its spectrums. (**c**) Generated triangular waveforms with repetition rates of 8, 10 and 12 GHz after 25 km SMF, and (**d**) its spectrums. (**e**) Generated sawtooth waveforms with repetition rates of 8, 10 and 12 GHz after 25 km SMF, and (**f**) its spectrums. The red, green, and blue curves represent the 8 GHz, 10 GHz, and 12 GHz waveforms respectively.

**Figure 9:**
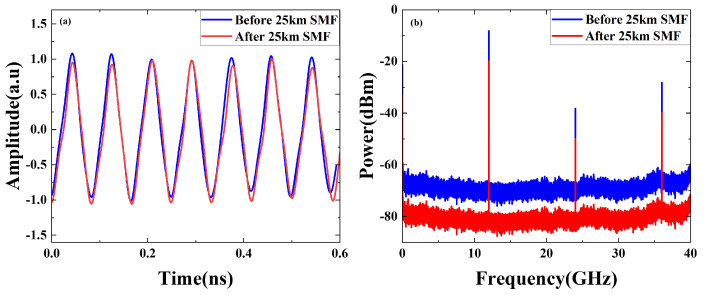
Experiment results. (**a**) Generated triangular waveforms before and after 25 km SMF, and (**b**) its spectrums.

The temporal waveforms of the generated triangular pulses with repetition rates of 8, 10 and 12 GHz and its electrical spectra correspond to Figure 8a,b. From electrical spectra, the power fading is approximately 10.84 dB, mainly attributed to insertion loss of SMF. Figure 8c and d show the time domain waveform and the electrical spectra of the generated rectangular waveforms of 8 GHz, 10 GHz and 12 GHz after passing through 25 km SMF. In addition, we validated the anti-dispersion capability of the system by generating sawtooth waveforms with varying frequencies. The temporal waveforms and electrical spectrum of the generated sawtooth waveforms at 8, 10 and 12 GHz under 25 km SMF transmission condition are depicted in Figure 8e,f. The power fading observed is approximately 12 dB. We also conducted a comparison between the original 12 GHz triangular waveform and the waveform after transmission through a 25 km optical fiber, both in the time domain and frequency domain. As illustrated in Figure 9a, the waveform maintains good performance after transmission. The measured spectra in Figure 9b indicate that the overall electrical power is reduced by approximately 11.2 dB, corresponding to a decrease in optical power of 5.6 dB, which fits well with the insertion loss of the SMF. Therefore, our proposed waveform generator can eliminate power fading due to dispersion over fiber transmission.

## 4. Conclusions

In this paper, a flexible anti-dispersion photonic microwave waveform generator with continuously tunable repetition rate based on DP-DPMZM has been theoretically and experimentally demonstrated. Two sub-MZMs are driven by the RF signals to generate the sidebands. By effectively manipulating the power of RF signals and precisely adjusting the modulation index of two sub-MZMs, it becomes feasible to regulate the power ratio between sidebands in accordance with diverse microwave waveforms. The results show that this method can successfully generate triangular waveforms, sawtooth waveforms and rectangular waveforms. The tunability of our signal generator has been validated through generation of these waveforms with repetition rates of 8, 10 and 12 GHz. In addition, the anti-dispersion characteristics of the system are deeply studied, and the optical waveforms are still complete after passing through 25 km fiber. Considering that the system has the ability to flexibly control the frequency and power of RF signals, this system gives us a new approach to realize the generation of arbitrary waveforms in theory and experiment, and provides a new solution for high-precision regulation and application in the future.

## Data Availability

The data presented in this study are available upon request from the corresponding author.
